# Supplementary material for: Spliceosome component Usp39 contributes to hepatic lipid homeostasis through the regulation of autophagy
Source: Nat Commun. 2023 Nov 3;14:7032. doi: 10.1038/s41467-023-42461-6 (PMC10624899; doi:10.1038/s41467-023-42461-6)
Supplement: Supplementary file 1 — Supplementary Information [file 41467_2023_42461_MOESM1_ESM.pdf]

Supplementary Information

Figure S1

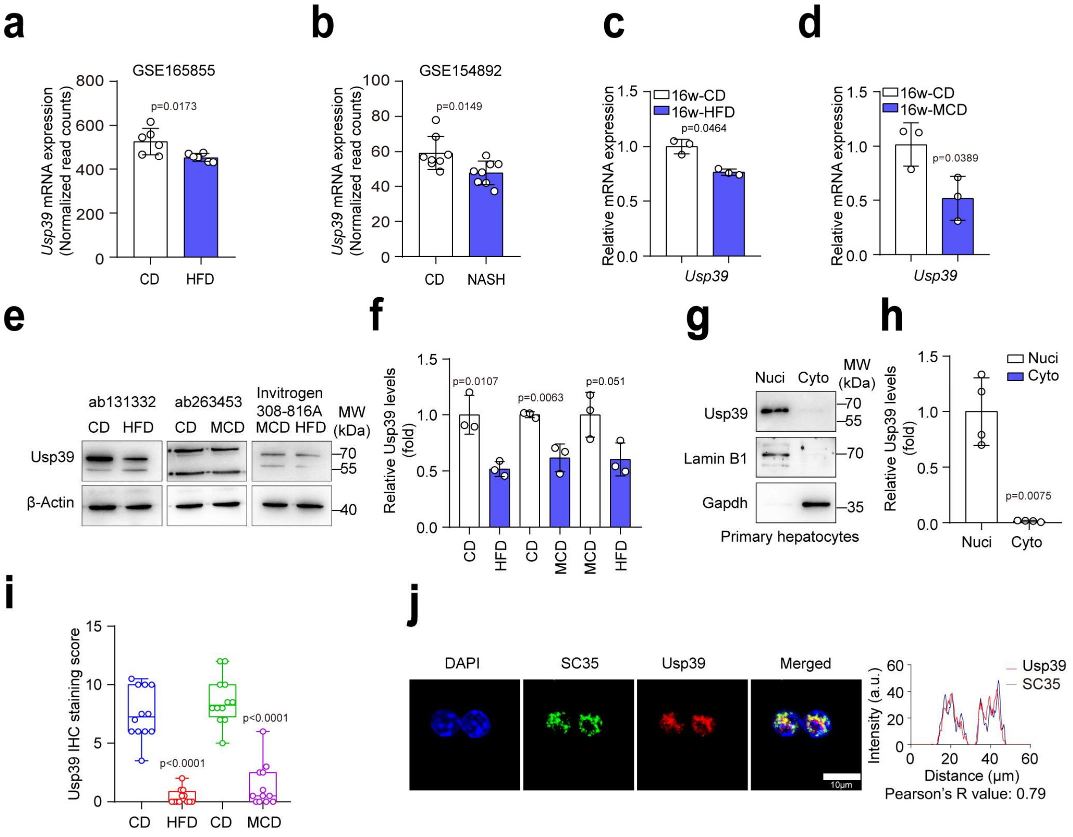

Figure S1. Usp39 expression and subcellular localization. a Usp39 expression was measured using transcriptomic data in the livers of chow-fed and HFD-fed mice from GEO database (GSE165855). **b** Usp39 expression was measured using transcriptomic data in the livers of chow-fed and CDAHFD-fed mice from GEO database (GSE154892). **c** mRNA expression analysis of liver *Usp39* gene in 16-week-old male mice fed CD or HFD for 12 weeks (n = 3 per group). **d** mRNA expression analysis of liver *Usp39* gene in 16-week-old male mice fed CD or MCD for 5 weeks (n = 3 per group). **e**, **f** Western blot results of Usp39 protein expression were detected using three primary antibodies. Usp39 protein expressions in livers of HFD-fed mice, MCD-fed mice, and chow-fed mice were shown (n = 3 per group) (e). Band intensity was quantified using Image J (**f**). **g**, **h** Usp39 protein levels in nuclei and cytoplasm from the livers

of 8-week-old mice primary cells were measured by immunoblotting (*n* = 4 per group). Band intensity was quantified using Image J. **i** Immunohistochemical (IHC) staining of Usp39 in the liver sections of MCD-fed and HFD-fed mice compared to those of the chow-fed mice (*n* = 12 per group). IHC image were quantified by pathologists. The bounds of the box were the upper and lower quartile with the median value in the center. The whiskers indicated the minima and maxima. The range from min to max show all points. **j** Immunofluorescence detection of Usp39 and SC35 co-localized in 8-week-old mouse primary cells (*n* = 4 per group) Scale bar, 10 μm. The quantification of co-localization was performed with Image J Coloc 2 and Plot Profile. Images are representative of at least three independent experiments. Result error bars showed mean ± S.D. Results were analyzed using unpaired two-sided Student's t-test. NS, stands for non-significant. HFD, high-fat diet. NASH, Non-alcoholic steatohepatitis. CD, control diet. MCD, methionine choline deficient diet. w, week. Nuci, nuclei. Cyto, cytoplasm. MW, molecular weight. Source data are provided as a Source Data file.

Figure S2

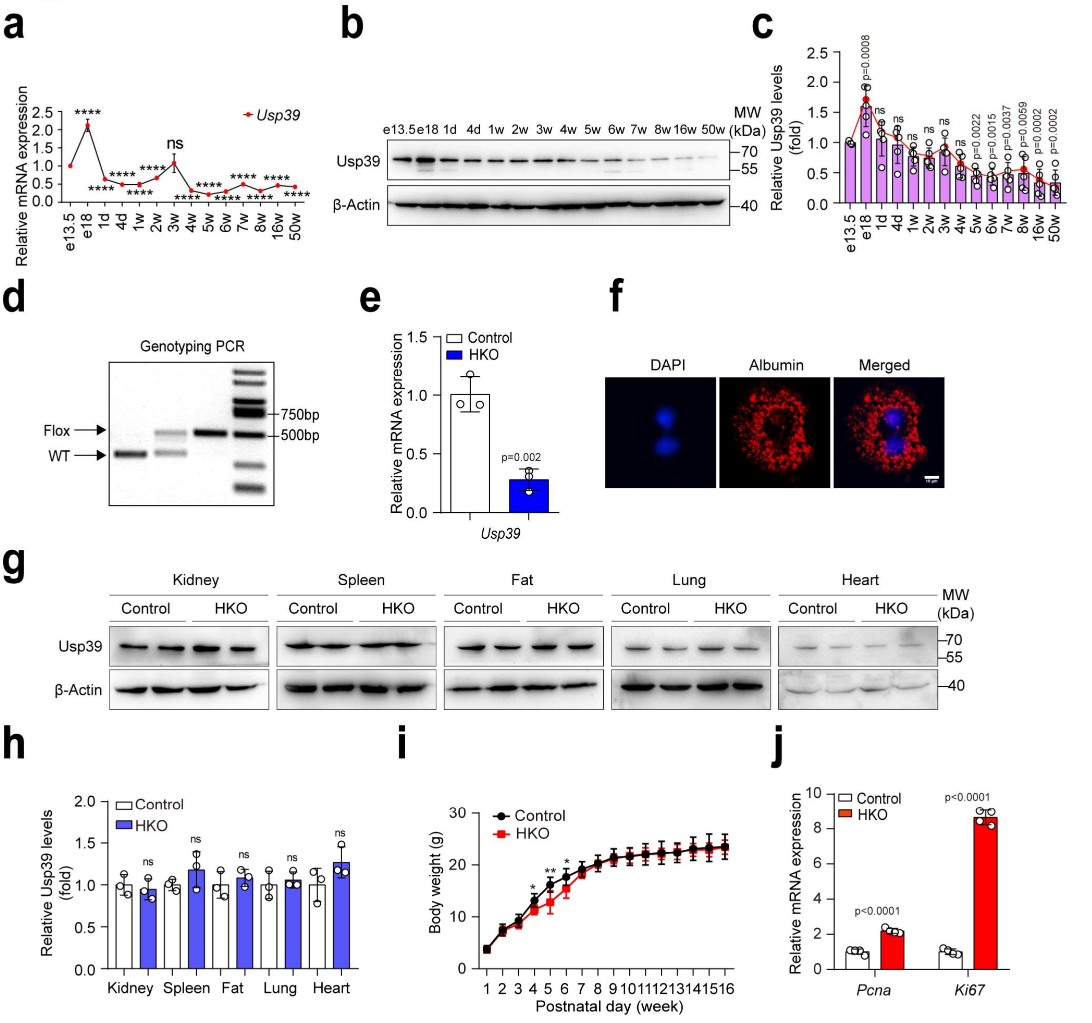

Figure S2. Generation of hepatocyte specific *Usp39* knockout mice and characterization of the phenotype. a, **b**, **c** qPCR ($n = 4$ per group) and western-blot analysis of Usp39 expression in the liver of WT mice at different ages (n = 5 independent samples). Band intensity was quantified using Image J. **d** Genotype identification of *Usp39*$^{f/+}$ and *Usp39*$^{f/f}$ mice using PCR analysis. **e** qPCR was performed to examine *Usp39* expression in livers of control and *Usp39*-HKO mice. **f** Immunofluorescence staining showed localization of albumin (red) and DAPI (blue) in primary hepatocytes. Image was captured by laser confocal microscope. Scale bar, 10 μm. **g**, **h** Western-blot analysis of Usp39 expression in various tissues of 5-week-old male mice (n = 3 per group). Band intensity was quantified using Image J. **i** Body weight measurement of

female mice from 1 to 16 weeks (n = 7 per group). **j** *Ki67* and *Pcna* mRNA expression was measured in the livers of control and *Usp39*-HKO male mice (n = 4 per group). Images are representative of at least three independent experiments. Result error bars showed mean ± S.D. Results were analyzed using unpaired two-sided Student's t-test and one-way analysis of variance (ANOVA) followed by Dunnett's multiple comparisons test. *P < 0.05, **P < 0.01, ****P < 0.0001, NS, stands for non-significant. MW, molecular weight. e, embryonic. d, day. w, week. Source data are provided as a Source Data file.

Figure S3

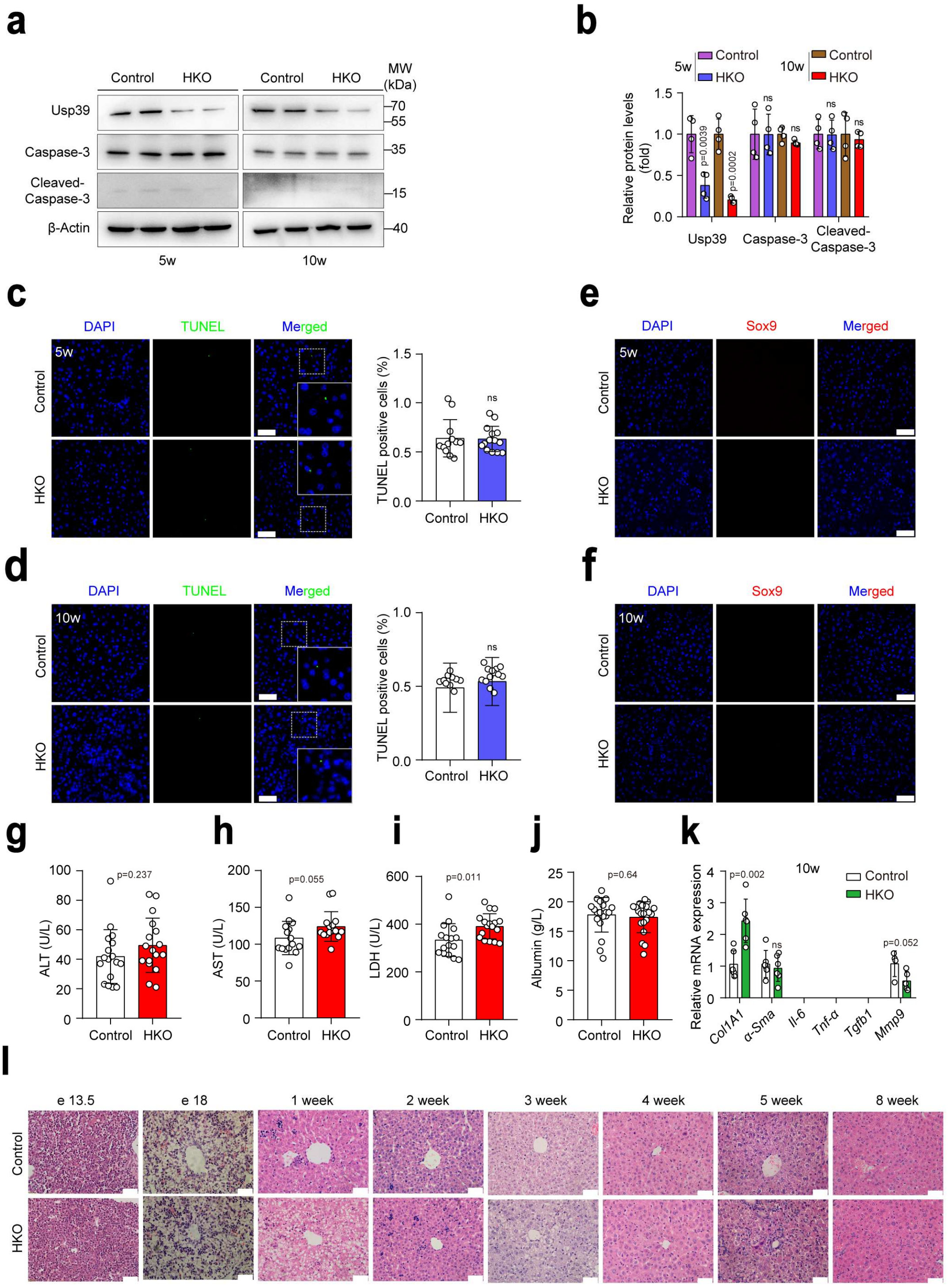

Figure S3. ***Usp39* knockout mice have slight liver injury.** **a**, **b** Immunoblotting was performed to determine caspase-3 and cleaved-caspase-3 expression in livers of 5- (n = 4 per group) and 10-week-old (n = 4 per group) control and *Usp39*-HKO male mice. Band intensity was quantified using Image J. **c**, **d** TUNEL staining on the liver sections of 5 (n = 12, 14 per

group) and 10-week-old (*n* = 11, 14 per group) control and *Usp39*-HKO male mice. Scale bars, 50 μm. **e**, **f** Immunofluorescence staining of Sox9 (red) and DAPI (blue) in liver tissue of 5- and 10-weeek-old mice (*n* = 4 per group). Scale bars, 50 μm. **g**, **h**, **i**, **j** Serum ALT (*n* = 17), AST (*n* = 15), LDH (*n* = 17), and albumin (*n* = 20) levels were measured in 10-week-old control and *Usp39*-HKO male mice fasted for 16h. **k** qPCR was performed to analyze mRNA expression of *Usp39* (*n* = 6)*, Col1a1* (*n* = 6)*, α-Sma* (*n* = 6)*, Il6* (*n* = 6)*, Tnf-α* (*n* = 6), *Tgfb1* (*n* = 6) and *Mmp9* (*n* = 4) in livers of 10-week-old control and *Usp39*-HKO male mice. **l** Representative images of H&E staining in the livers of control and *Usp39*-HKO male mice at various developmental stages fasted for 16 h (*n* = 3 per group). Scale bar, 50 μm. Images are representative of at least three independent experiments. Result error bars showed mean ± S.D. Results were analyzed using unpaired two-sided Student's t-test. NS, stands for non-significant. MW, molecular weight. e, embryonic. h, hours. w, week. Source data are provided as a Source Data file.

Figure S4

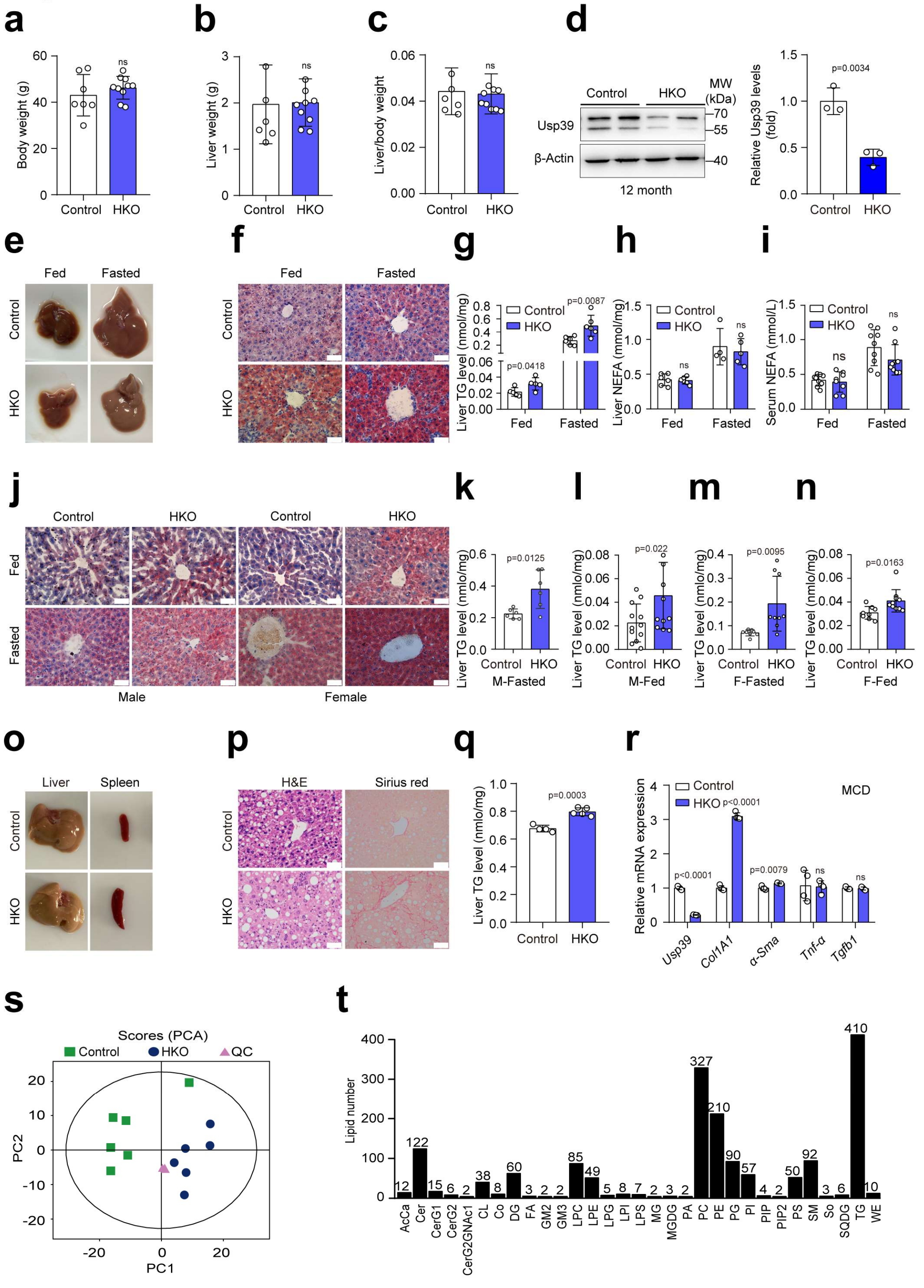

Figure S4. Hepatocyte-specific *Usp39* deletion induces lipid accumulation and fibrosis.

a, **b**, **c** Statistical analysis of body weight, liver weight and liver/body weight ratio of 12-month-

old male mice (control *n* = 7 per group, *Usp39*-HKO *n* = 10 per group). **d** Usp39 expression in the liver of 12-month-old male mice (*n* = 3 per group). **e** Representative liver images of 5-week-old male mice fed or fasted for 16 h. **f** Oil Red O staining of control and *Usp39*-HKO male mice fed or fasted for 16 h (*n* = 3 per group). Scale bars, 50 µm. **g**, **h** Hepatic triglyceride levels and hepatic nonesterified fatty acids (NEFA) were measured in male mice fed (*n* = 5) or fasted for 16 h (*n* = 4, 6 per group). **i** Concentrations of non-esterified fatty acids in male mice serum that were fed or fasted for 16h (*n* = 8, 9). **j** Oil red O staining of the liver section from 10-week-old male and female mice fasted for 16 h (*n* = 3 per group). Scale bar, 50 µm. **k**, **l**, **m**, **n** Hepatic triglyceride level from 10-week-old male and female mice that fed or fasted for 16 h (*n* = 6-12). **o** Appearance of liver (left) and spleen (right) of 16-week-old male mice fed for 5 weeks by MCD (*n* = 3). **p** H&E staining (left) and Sirius Red staining (right) from 16-week-old male mice liver sections fed for 5 weeks by MCD (*n* = 3). Scale bar, 50 µm. **q** Hepatic triglyceride level from 16-week-old male mice fed for 5weeks by MCD (*n* = 4, 5 per group). **r** mRNA expression analysis of *Usp39*, *Collagen1A1*, *α-Sma, Tnf-α* and *Tgfb1* in livers of control and *Usp39*-HKO male mice fed with MCD for 5 weeks (*n* = 4 per group). **s** PCA (Principal component analysis) analysis of ion peaks after Pareto-scaling extracted from experimental and QC samples. **t** Number of the subclass of lipids based on lipidomic data in liver samples of 5-week-old control and *Usp39*-HKO mice fasted for 16 h (*n* = 6 per group). Images are representative of at least three independent experiments. Result error bars showed mean ± S.D. Results were analyzed using unpaired two-sided Student's t-test. NS, stands for non-significant. h, hours. Source data are provided as a Source Data file.

Figure S5

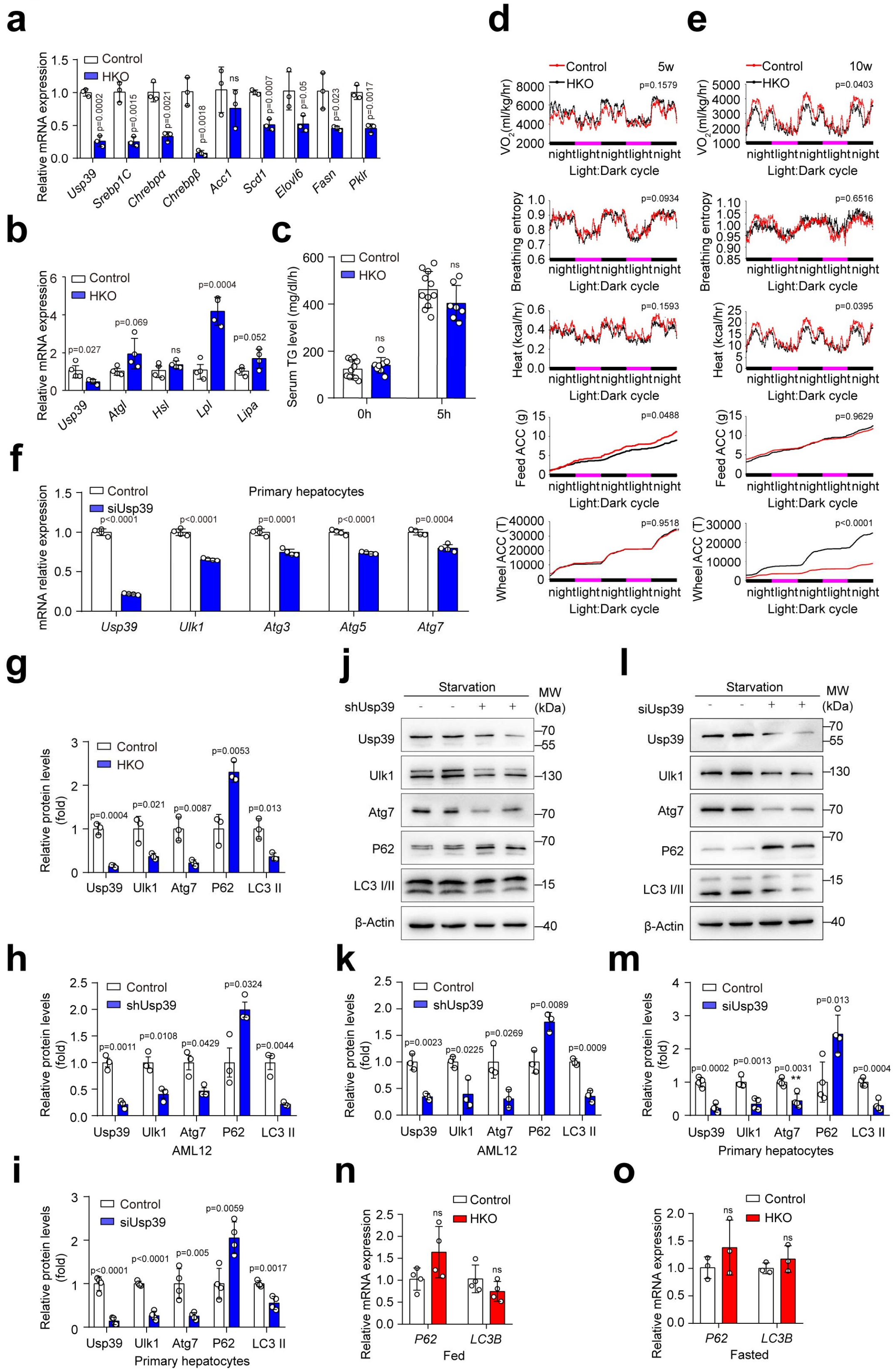

Figure S5. Mechanism study of excessive hepatic lipid accumulation upon loss of Usp39.

a, **b** mRNA expression analysis of lipogenesis and lipolysis related genes (*n* = 3 per group). **c** Triglyceride secretion was measured in male mice after injected with poloxamer 407, an inhibits lipoprotein lipase activity (0 h *n* = 11 per group, 5 h *n* = 11, 10 per group). **d**, **e** Oxygen consumption, RER (breathing entropy), energy expenditure, food intake and locomotor activity were measured using metabolic cages in 5-week-old (**d**) (*n* = 6, 7) and 10-week-old (**e**) (*n* = 7) male mice. **f** qPCR was performed to analyze expression of autophagy-related genes in *Usp39* knockdown and control primary cells (*n* = 4 per group). **g** Immunoblotting was performed to analyze protein levels of autophagy-related genes in 5-week-old male mice (*n* = 3 per group) fasted for 16 h. Band intensities were quantified by Image J. **h** Immunoblotting was performed to analyze protein levels of autophagy-related genes in *Usp39* knockdown and control AML12 cells supplemented with 0.4 mM oleic acid for 6 h (*n* = 3 per group). Band intensities were quantified by Image J. **i** Immunoblotting was performed to analyze protein levels of autophagy-related genes in *Usp39* knockdown and control primary cells supplemented with 0.4 mM oleic acid for 6 h (*n* = 4 per group). Band intensities were quantified by Image J. **j**, **k**, **l**, **m** AML12 (*n* = 3) and primary hepatocytes (*n* = 4) were transfected with shUsp39, siUsp39 and control vectors. After 72 h, cells were cultured in serum-free medium for 2 h. Immunoblotting was performed to measure protein levels of Usp39, Ulk1, Atg7, P62 and LC3I/II. Band intensities were quantified by Image J. **n**, **o** qPCR was performed to analyze expression of *P62* and *LC3B* of male mice fed (*n* = 4) or fasted for 16 h (*n* = 3). Images are representative of at least three independent experiments. Result error bars showed mean ± S.D. Results were analyzed using unpaired two-sided Student's t-test or two-way analysis of variance (ANOVA) (**d**, **e**). NS, stands for non-significant. w, week. h, hours. Source data are provided as a Source Data file.

Figure S6

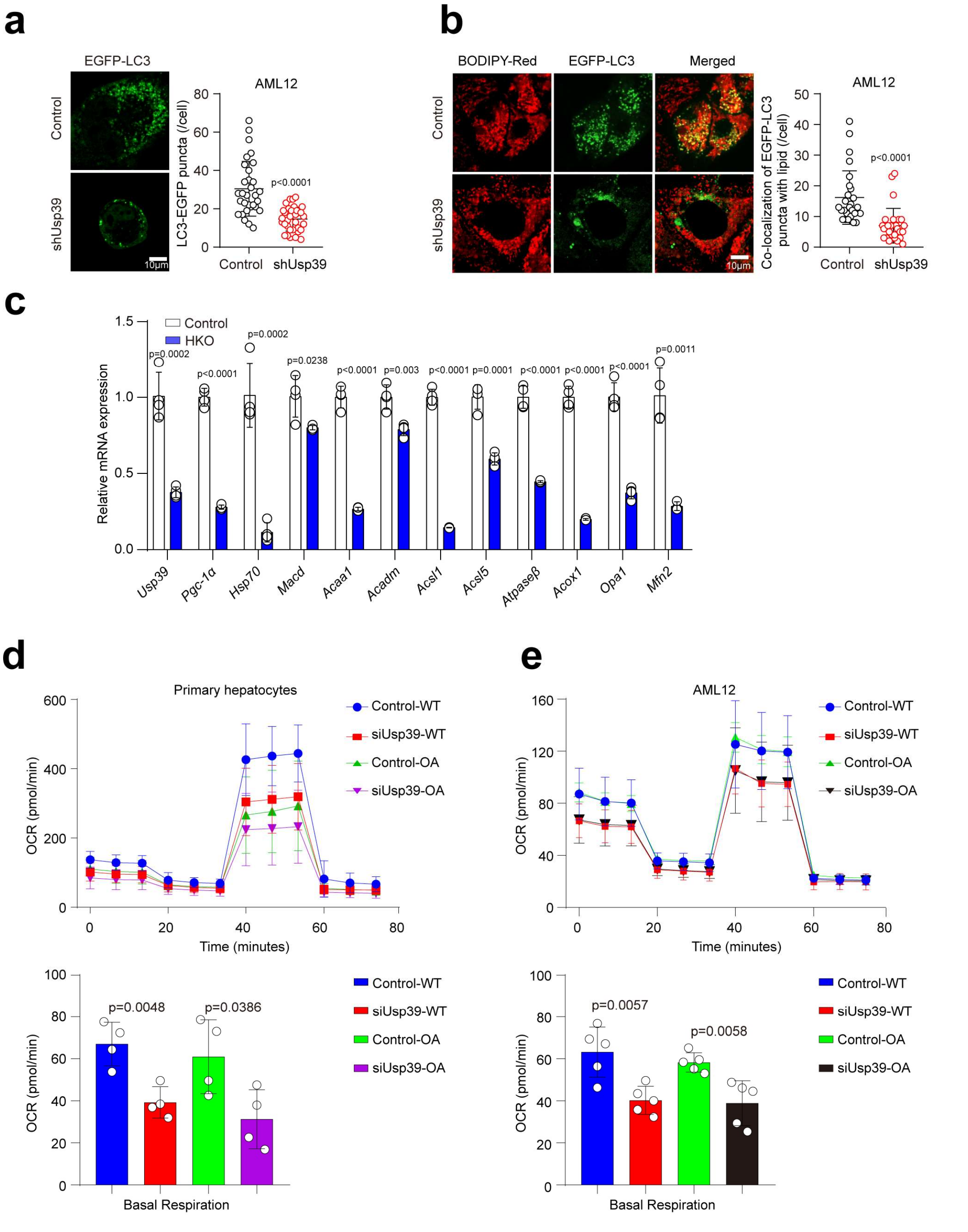

Figure S6. Autophagic degradation of lipid droplet and FAO is blocked upon *Usp39* knockout. a AML12 cells were transfected with Ad-EGFP-LC3, shUsp39 and control vectors. After 72 h, cells were cultured in serum-free for 2 h. Fluorescent images were captured and the average number of EGFP-LC3 puncta/cell were count and analyzed ($n = 31$ cells for control, $n = 36$ cells for shUsp39). Scale bars, 10 μm. **b** AML12 cells were transfected with Ad-EGFP-

LC3, shUsp39 and control vectors. After 72 h, cells using medium supplemented with 0.4 mM oleic acid for 6 h. BODIPY C_{12} staining of lipid droplets in AML12 cells. Co-localization of EGFP-LC3 puncta and BODIPY C_{12} were shown in the merged image (*n* = 27 cells for control, *n* = 28 cells for shUsp39). Scale bars, 10 μm. **c** qPCR was performed to analyze mRNA expression of fatty acid oxidation genes in *Usp39* -HKO and control mice (*n* = 4 per group). **d**, **e** Primary hepatocytes and AML12 were transfected with siUsp39 and control vectors for 72 h, cells were treated with medium supplemented with 0.4 mM oleic acid for 6 h. Oxygen consumption rate (OCR) was detected as a maker for oxidative phosphorylation (OXPHOS) levels by seahorse (*n* = 6 per group). Images are representative of at least three independent experiments. Result error bars showed mean ± S.D. Results were analyzed using unpaired two-sided Student's t-test. NS, stands for non-significant. h, hours. Source data are provided as a Source Data file.

Figure S7

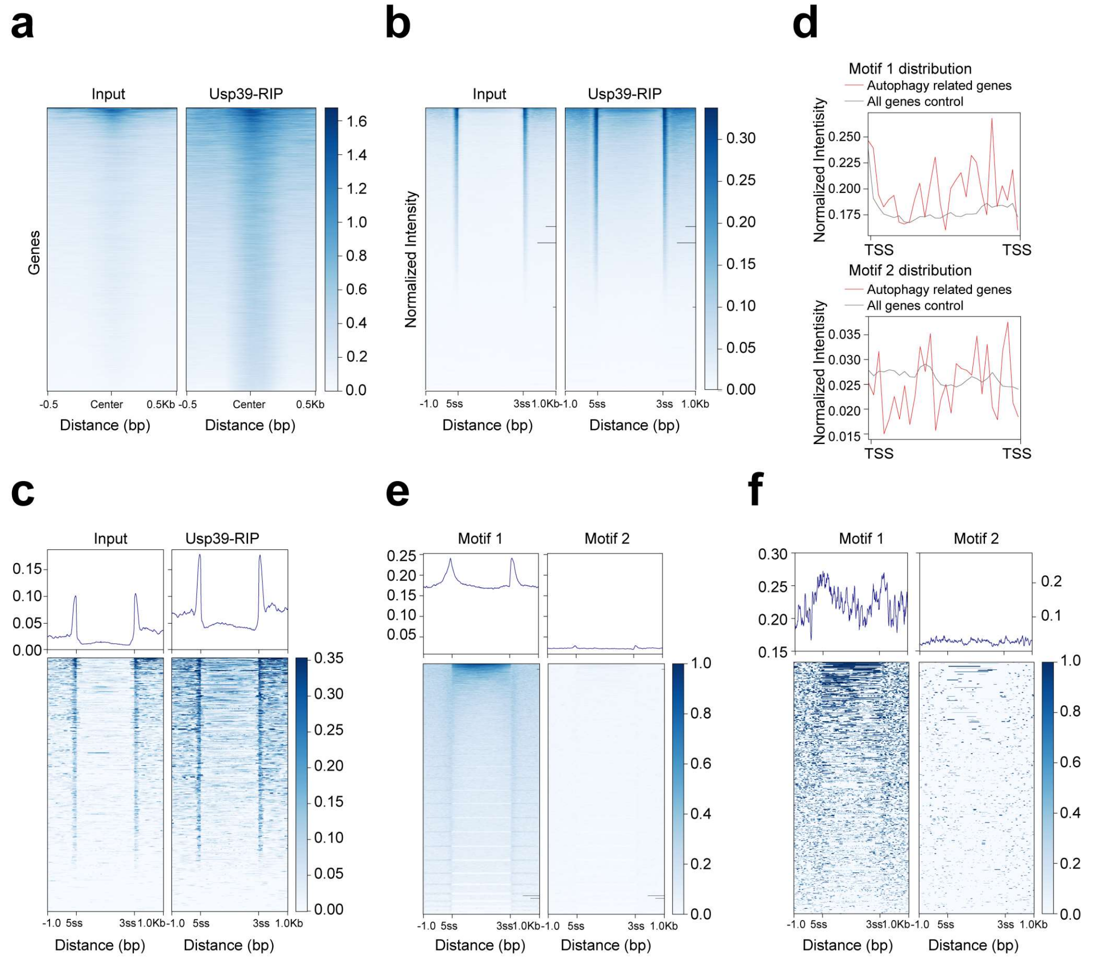

Figure S7. Identification of the genome-wide Usp39 binding region on RNAs. a Input and Usp39-RIP signal intensity around the Usp39 binding sites were identified by Piranha based on RIP-seq data. **b** Normalized reads intensity of Usp39 RIP-seq data around 5' and 3' splicing sites. **c** Normalized reads intensity of Usp39 RIP-seq data around 5' and 3' splicing sites of autophagy-related genes. **d** Usp39 binding Motif 1 and Motif 2 distribution from transcription start sites (TSS) to transcription termination sites (TTS) of autophagy-related targets. **e** Distribution of Usp39 binding Motif 1 and Motif 2 at all splice sites of mouse genome. Motif 1 is enriched in 5' and 3' splicing sites. **f** Distribution of Usp39 binding Motif1 and Motif2 at splice sites of autophagy-related genes. Motif 1 is enriched in 5' and 3' splicing sites of autophagy-related targets. Source data are provided as a Source Data file.

Figure S8

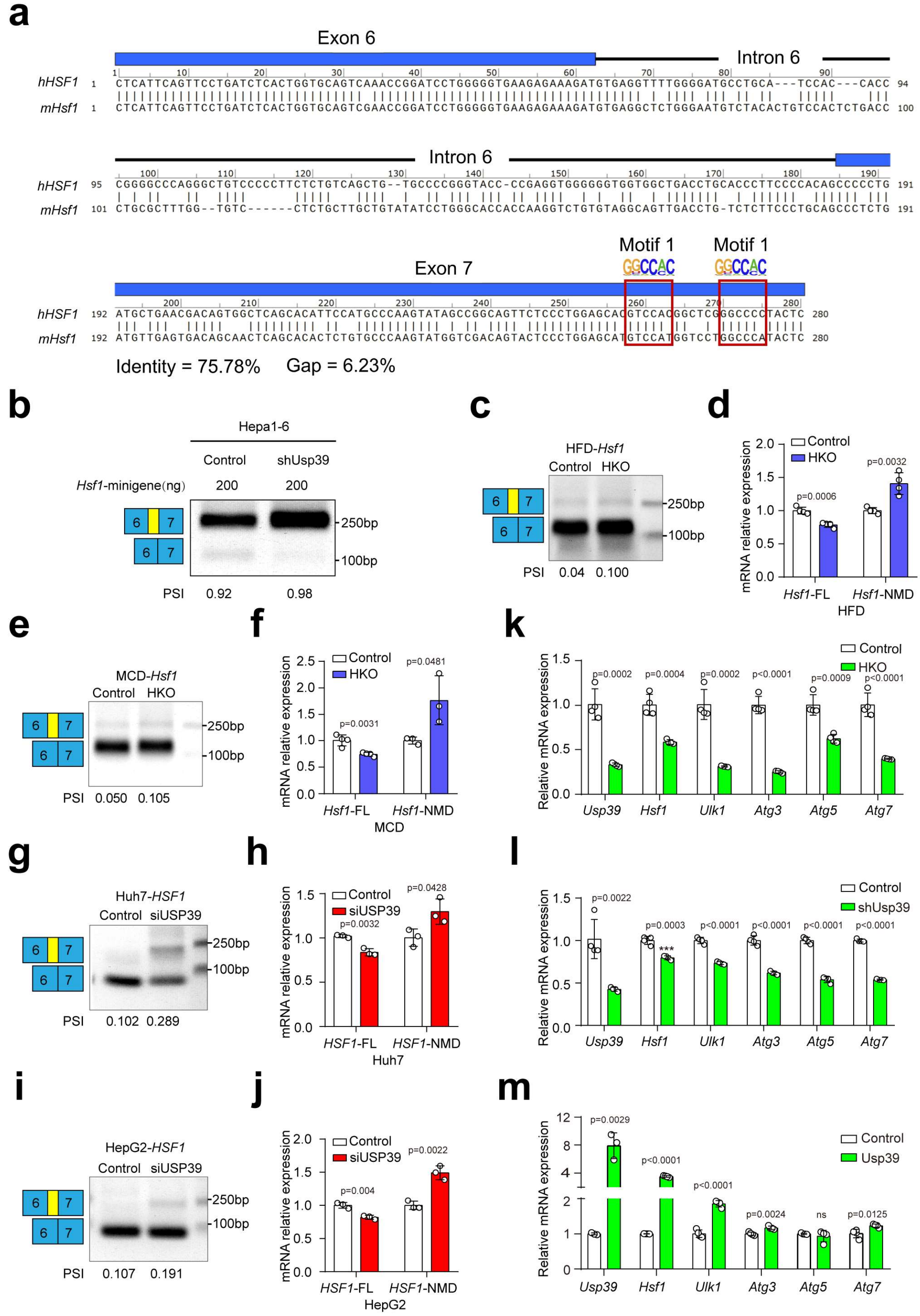

Figure S8. ***Usp39*** **deletion leads to mis-splicing and fast degradation of** ***Hsf1*** **in hepatocytes.**

a The homologous sequences of human *HSF1* were compared with mouse *Hsf1*. The alignment results near exon 6, intron 6, and exon 7 of mouse *Hsf1* gene were shown. Usp39 binding Motif

1 near the alternative region was indicated with red rectangles. **b** Semi-quantitative RT-PCR gel images showing expression of *Hsf1*-minigene transcripts in Hepa1-6 cells transfected with *Hsf1*-minigene and shUsp39 vector. **c**, **d** Semi-quantitative RT-PCR and qPCR (*n* = 4 per group) was performed to validate AS events in Hsf1 of 16-week-old control and *Usp39*-HKO male mice fed the HFD for 12 weeks. Percent spliced in (PSI) was quantified. **e**, **f** Semi-quantitative RT-PCR and qPCR (*n* = 4 per group) was performed to validate AS events in Hsf1 of 16-week-old control and *Usp39*-HKO male mice fed the MCD for 5 weeks. Percent spliced in (PSI) was quantified. **g**, **h**, **i**, **j** Semi-quantitative RT-PCR and qPCR was performed to validate AS events in *HSF1* in Huh7 and HepG2 cells transfected with control and siUSP39 interfering RNA after 48 h (*n* = 3 independent experiments). Images are representative of at least three independent experiments. **k** Autophagy-related genes were measured in 5-week-old control and *Usp39*-HKO mice that were fasted for 16 h (*n* = 4 per group) by qPCR. **l**, **m** qPCR was performed to analyze autophagy-related genes in AML12 cells with *Usp39* knockdown or overexpression (*n* = 4 per group). Images are representative of at least three independent experiments. Result error bars showed mean ± S.D. Results were analyzed using unpaired two-sided Student's t-test. NS, stands for non-significant. h, hours. Source data are provided as a Source Data file.

Figure S9

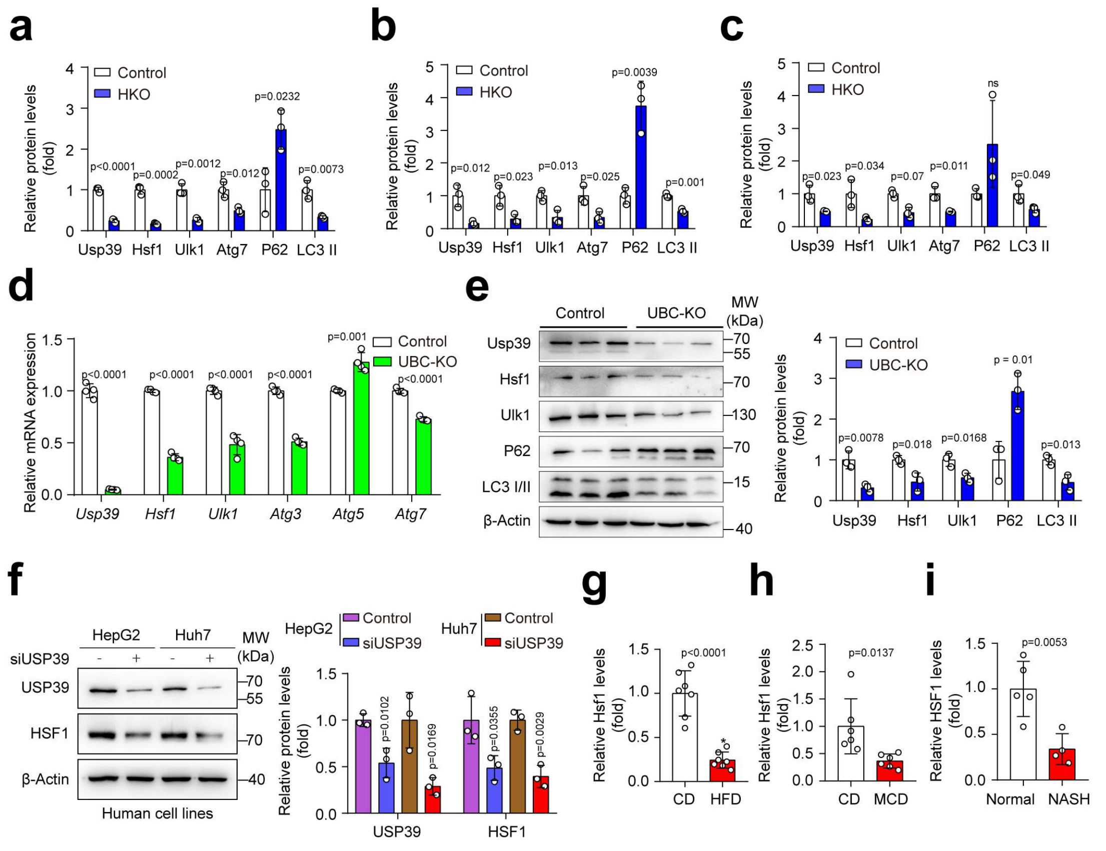

Figure S9. *Usp39* deletion leads to decreased expression of Hsf1 in mice and human hepatocytes lines. a-c Immunoblotting was performed to measure the protein levels of Usp39, Hsf1 and autophagy-related genes in male mice under fed (Fig.6**i**), fasted (Fig.6**j**) and HFD (Fig.6**k**) conditions (*n* = 3 per group). Band intensities were quantified by Image J. **d, e** *Usp39*$^{fl/fl}$ mice were crossed with UBC-Cre-ERT2 mice to generate *Usp39*$^{fl/fl}$; UBC-Cre^{ERT2} mice. After 10 weeks, tamoxifen was injected intraperitoneally for three days and the drug release was one day. Drug-induced Cre mediated the recombination will result in deleting of *Usp39*-floxed sequence in whole body cells/tissues. qPCR (*n* = 4 per group) and immunoblotting (*n* = 3 per group) were performed to measure the expression of autophagy-related genes in control and *Usp39*$^{fl/fl}$; UBC-Cre^{ERT2} mice (*Usp39*UKO mice). Band intensities were quantified by Image J. **f** Immunoblotting analysis of Hsf1 in HepG2 and Huh7 cells transfected with control and

siUSP39 for 72 h (*n* = 3 per group). Band intensities were quantified by Image J. **g**, **h** Hepatic expression of Hsf1 proteins in MCD-fed (*n* = 6 per group) and HFD-fed (*n* = 7 per group) mice compared to those of the chow-fed mice. Band intensities were quantified by Image J. **i** HSF1 protein was measured in human healthy (*n* = 5) and NASH (*n* = 4) livers. Band intensities were quantified by Image J. Images are representative of at least three independent experiments. Result error bars showed mean ± S.D. Results were analyzed using unpaired two-sided Student's t-test. NS, stands for non-significant. h, hours. Source data are provided as a Source Data file.

Figure S10

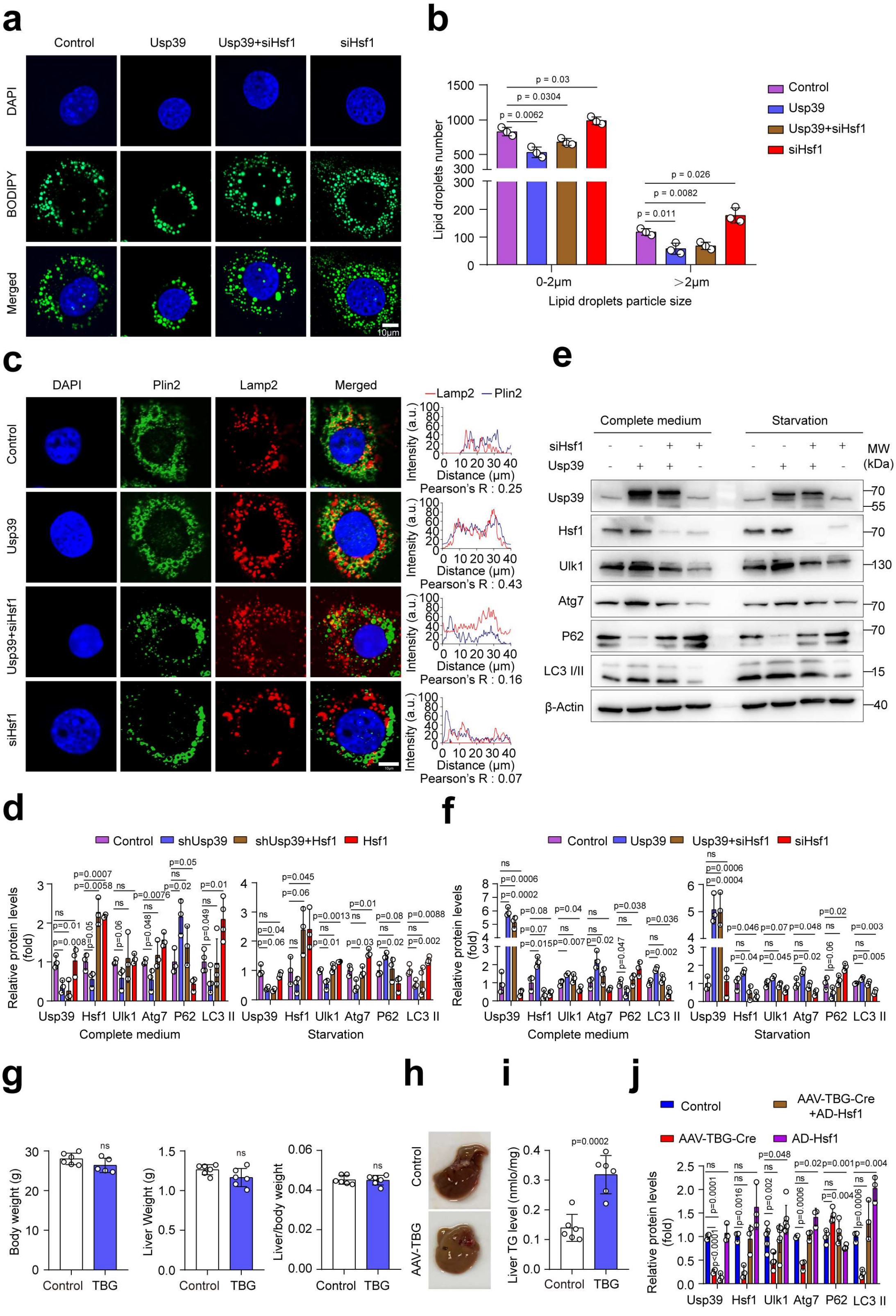

Figure S10. Hsf1 promotes autophagy and alleviates hepatic steatosis caused by Usp39 depletion. a, **b** BODIPY staining of lipid droplets in AML12 cells transfected with Usp39

overexpression vector or siRNAs targeting Hsf1. Cells were then cultured in medium supplemented with 0.4 mM oleic acid for 6 h followed by serum deprivation for 2 h. Fluorescent images were captured, BODIPY was shown in green, Image J software was used to counted the lipid droplet number and size of 30 BODIPY-stained cells in each group (*n* = 3 independent experiments). Scale bars, 10 µm. **c** Immunofluorescence staining of Lamp2 (red) and Plin2 (green) in AML12 cells transfected with Usp39 overexpression vector or siRNAs targeting Hsf1 using medium supplemented with 0.4 mM oleic acid for 6 h followed by serum-deprivation for 2 h. Scale bars, 10 µm. Co-localization was analyzed by Image J software. **d** Immunoblotting band (Fig. 7d) intensities were quantified by Image J (*n* = 3,4 per group). **e**, **f** Immunoblotting was performed to measure protein levels of Usp39, Hsf1 and autophagy-related genes in AML12 cells transfected with Usp39 overexpression vector or siRNAs targeting Hsf1 in complete or serum deprived medium (*n* = 3 per group). Band intensities were quantified by Image J, showing in Fig. S10**f**. **g** Body weight, liver weight and liver/body weight ratio were analyzed in 12-week-old control and AAV-TBG-Cre male mice fasted for 16 h (*n* = 6 per group). **h** Appearance of livers of 12-week-old control and AAV-TBG-Cre male mice. **i** Hepatic triglyceride level was measured in 12-week-old AAV- empty and AAV-TBG-Cre male mice fasted for 16 h (*n* = 6 per group). **j** Immunoblotting band (Fig. 7**g**) intensities were quantified by Image J (*n* = 3, 4, 6 per group, from three independent experiments). Images are representative of at least three independent experiments. Result error bars showed mean ± S.D. Results were analyzed using unpaired two-sided Student's t-test. NS, stands for non-significant. h, hours. Source data are provided as a Source Data file.

Supplementary Table 1. Clinicopathological characteristic

Correlation between USP39 expression and clinicopathological characteristics

Clinicopathological Feature	Number	USP39 expression		*p* value
		Low	High	
**Age (years)**				
<60	114	66	48	p = 0.0197
≥60	137	48	89	
**Fibrosis stage**				
0-1	78	30	48	p = 0.0231
2-4	193	104	89	
**NAFLD activity score**				
<3	74	22	52	p<0.0001
≥3	197	112	85	
**Gender**				
Female	116	61	55	p = 0.3920
Male	155	73	82	
**PLS-NAFLD-based HCC risk prediction**				
Low risk	80	34	46	p = 0.5729
High risk	133	62	71	

The chi-square test was used to analyze the differences in clinical characteristics. *p < 0.05

Supplementary Table 2. Sequence information

Genotype-PCR primers		
Name	Forward primer 5'-3'	Reverse primer 5'-3'
Usp39	CATCTCACTTGCCTGGATTCC	CTCCAGCATCATAAATGGAGGAC
Cre	CCCGCAGAACCTGAAGATG	GACCCGGCAAAACAGGTAG
**RT-PCR primers**		
Name	Forward primer 5'-3'	Reverse primer 5'-3'
Usp39	GTCACTGCCCGTACTTGGATA	GTATGCGTTGATGTGCGAGAG
Ki67	AACGGGGTTACTATAGATGAGCCTG	CTGGAAATTCTGTTGGCTTGCTTC
Pcna	TTGCACGTATATGCCGAGACC	GGTGAACAGGCTCATTCATCTCT
Alb	GAAGTGGGTAACCTTTCTCC	ACAGCAGTCAGCCAGTTCACC
H19	CAACATCCCACCCACCGTAA	GCTCACCAAGAAGGCTGGAT
Afp	CTTCCCTCATCCTCCTGCTAC	ACAAACTGGGTAAAGGTGATGG
Collagen1a1	TCACCTACAGCACCCTTGTG	GGTGGAGGGAGTTTACACGA
α-Sma	GGAGAAGCCCAGCCAGTCGC	AGCCGGCCTTACAGAGCCCA
Tgfb1	TTGCTTCAGCTCCACAGAGA	TGGTTGTAGAGGGCAAGGAC
Tnf-α	CATCTTCTCAAAATTCGAGTGACAA	TGGGAGTAGACAAGGTACAACCC
Ulk1	ACCATTGTCTACCAGTGT	AGTGTCTTGTTCTTCTCATAA
Atg3	TCACAACACAGGTATTACAG	CTTCCTCGTCTTCTTCATC
Atg5	TGTGCTTCGAGATGTGTGGTT	ACCAACGTCAAATAGCTGACTC
Atg7	CAGAAGAAGTTGAACGAGTA	CAGAGTCACCATTGTAGTAAT
Il6	AGTTGCCTTCTTGGGACTGA	TCCACGATTTCCCAGAGAAC
Mmp9	CGTCGTGATCCCCACTTACT	AACACACAGGGTTTGCCTTC
P62	CTGAAGAATGTGGGGGAGAG	TTTCTGGGGTAGTGGGTGTC
LC3B	TTATAGAGCGATACAAGGGGGAG	CGCCGTCTGATTATCTTGATGAG
Srebp1C	GGAGCCATGGATTGCACATT	GGAAGTCACTGTCTTGGTTGTTGA
Chrebpα	CGACACTCACCCACCTCTTC	TTGTTCAGCCGGATCTTGTC
Chrebpβ	TCTGCAGATCGCGYGGAG	CTTGTCCCGGCATAGCAAC
Acc1	TGAATCTCACGCGCCTACTATG	ATGACCCTGTTGCCTCCAAAC
Scd1	TTCTTGCGATACACTCTGGTGC	CGGGATTGAATGTTCTTGTCGT
Elovl6	TGCCATGTTCATCACCTTGT	TGCTGCATCCAGTTGAAGAC
Fasn	AAGTTGCCCGAGTCAGAGAA	CGTCGAACTTGGAGAGATCC
Pklr	GAACATTGCACGACTCAACTTC	CAGTGCGTATCTCGGGACC
Atgl	CCAACGCCACTCACATCTAC	GCCTCCTTGGACACCTCAAT
Hsl	ACGCTACACAAAGGCTGCTT	TCTCGTTGCGTTTGTAGTGC
Lpl	TGGCCCATAGCAGGTCT	AAGGCCAGGTGTTTCAATC
Lipa	GAACACTCGGTCCTGACAGGAGAT	AAGCCGTGCTGAAGATACACAACT
Gapdh	AGGTCGGTGTGAACGGATTTG	TGTAGACCATGTAGTTGAGGTCA
Actb	GTGACGTTGACATCCGTAAAGA	GCCGGACTCATCGTACTCC
Hsf1-qPCR	GGGAAACAGGAGTGTATGGACT	CTTGTTGACAACTTTTTGCTGCT
Hsf1-NMD	CTCACTGGTGCAGTCGAACC	GGACAGTGTAGACATTCCCAGA
Hsf1-FL	TGACAGCAACTCAGCACACTC	TGCTCCAGGGAGTACTGTCG
Pgc1α-F	ACCATGACTACTGTCAGTCACTC	GTCACAGGAGGCATCTTTGAAG

Hsp70	TGGTGCTGACGAAGATGAAG	AGGTCGAAGATGAGCACGTT
Macd	ATGACGGAGCAGCCAATGAT	TCGTCACCCTTCTTCTCTGCTT
Acaa1	TCTCCAGGACGTGAGGCTAAA	CGCTCAGAAATTGGGCGATG
Acadm	GCGAGCAGAAATGAAACTCC	AGCTCTAGACGAAGCCACGA
Acsl1	TGGGGTGGAAATCATCAGCC	CACAGCATTACACACTGTACAACGG
Acsl5	TCCCAGCCCACCTCTGATGATGTG	ACAAACTGTCCCGCCGAATG
Atpaseβ	GGTTCATCCTGCCAGAGACTA	AATCCCTCATCGAACTGGACG
Acox1	GGATGGTAGTCCGGAGAACA	AGTCTGGATCGTTCAGAATCAAG
Opa1	TGGAAAATGGTTCGAGAGTCAG	CATTCCGTCTCTAGGTTAAAGCG
Mfn2	AGAACTGGACCCGGTTACCA	CACTTCGCTGATACCCCTGA
**RIP-PCR primers**		
Name	Forward primer 5'-3'	Reverse primer 5'-3'
RIP-PCR- Hsf1-E5-E6	CCAGCAGCAAAAAGTTGTCA	CTGCACCAGTGAGATCAGGA
RIP-PCR- Hsf1-E6-E7	ATCCTGGGGGTGAAGAGAAA	GGCACAGAGTGTGCTGAGTT
RIP-PCR- Hsf1-E7	TGAGTGACAGCAACTCAGCA	TGCTCCAGGGAGTACTGTCG
RIP-PCR-Hsf1-E7-E8	TGAGTGACAGCAACTCAGCA	TGAGTGACAGCAACTCAGCA
RIP-PCR-U6 snRNA	CGCTTCGGCAGCACATATAC	TTCACGAATTTGCGTGTCAT
RIP-PCR-Ptbp1-E4	GTCCCTTCCAGAGTCATCCA	CATCTCAATGAAGGCCTGGT
**Semiquantitative RT-PCR primers**		
Name	Forward primer 5'-3'	Reverse primer 5'-3'
Ulk3	AATCAGGCCCTGCTAAGACA	GGAGGCGTGGTTTGTCAC
Nrbp2	AGCCTCAAGCAGTTCCTCAA	GTGCAGACAGGATCTGCGTA
Hsf1-Sei-FL	TGAGAAAGATCCCTCTGATGTTG	CGACCATACTTGGGCACAG
Hsf1-Sei-NMD	TCTCACTGGTGCAGTCGAAC	GCCCAGGATATACAGCAAGC
Trp53inp1	TCTCAGTGAGGCGAGTTGTG	CGACGGAGACCATTTCTGTT
Tcirg1	GTGGCAGCTGACCATACTGA	CTTGTAGGGCTCCACAGCA
**RNA-pull down Primers**		
Name	Forward primer 5'-3'	Reverse primer 5'-3'
Hsf1-E5-I7	TAATACGACTCACTATAGGGGAGAACGAGGCCCTGTGG	TGGGAGCCAGCTCAGTGATA
**Primers for plasmid construction**		
Name	Forward primer 5'-3'	Reverse primer 5'-3'
Usp39-pT3G-3FPS	ACGAAGGGCGGAGGAGGATCCATGTCTAGCCGGTCCAAGCG	CCGTCATAGCGCGTTAATTAATCAAGCCCCCTGCTGGTT
Hsf1-pLenti- C-Myc-DDK-IRES-Puro	AGGAGATCTGCCGCCGCGATCGCATGGATCTGGCCGTGGGC	CTCGAGCGGCCGCGTACGCGTGGAGACAGTGGGGTCCTTGG
**siRNA sequence**		
Name	Sequence (5′ - 3′)	Manufacturer

siNC	UUCUCCGAACGUGUCACGUTT	Guangzhou RiboBio Co., Ltd.
siUpf1#1	GAUGCAGUUCCGCUCCAUU	Guangzhou RiboBio Co., Ltd.
siUpf1#2	AGAGCGCATTGAAAACGTT	Guangzhou RiboBio Co., Ltd.
siUsp39#1	GCATCAAGAGATTTACTAA	Guangzhou RiboBio Co., Ltd.
siUsp39#2	CAAGTGGTATGAATTACAA	Guangzhou RiboBio Co., Ltd.
siHsf1 #1	CCTGAAGAGTGAGGACATA	Guangzhou RiboBio Co., Ltd.
siHsf1 #2	GGACACAACCGGAGCCCAA	Guangzhou RiboBio Co., Ltd.
**shRNA targeting sequence**		
Name	Sequence (5′ - 3′)	Manufacturer
PLKO.1-shUsp39#2	TRCCCTGACAACTATGAAATCATT	Jinan WZ Biosciences INC
PLKO.1-shUsp39#4	CCCGCTCTATAAGGATGAGAA	Jinan WZ Biosciences INC
**Human Semiquantitative RT-PCR primers**		
Name	Forward primer 5'-3'	Reverse primer 5'-3'
hHSF1-semi	CTCACTGGTGCAGTCAAACC	GAACTGCCGGCTATACTTGG
**Human RT-PCR primers**		
hHSF1- FL	AGAGAAAGATCCCCCTGATG	GGCTATACTTGGGCATGGAA
hHSF1-NMD	GTGAGGTTTTGGGGATGC	GTGCAGGTCAGCCACCA
**Human siRNA sequence**		
sihUSP39#1	TCAAGAGATTCACTAAGAA	Guangzhou RiboBio Co., Ltd.
sihUSP39#2	GCAGTTGTACTTTGCAGTA	Guangzhou RiboBio Co., Ltd.

Note: Primer sequences are all produced by Sangon Biotech (Shanghai) Co., Ltd., unless the manufacturer is specifically indicated in the table.

Supplementary Table 3. Key resources table

REAGENT or RESOURCE	SOURCE	IDENTIFIER
Antibodies		
Rabbit monoclonal anti-Usp39	Abcam	ab131244<br>RRID:AB_11155482
Rabbit monoclonal anti- LC3A/B (D3U4C) XP	Cell Signaling Technology	12741<br>RRID:AB_2617131
Rabbit polyclonal anti- Albumin	Proteintech	16475-1-AP<br>RRID:AB_2242567
Mouse monoclonal anti-Actin	Proteintech	66009-1-Ig<br>RRID:AB_2687938
Rabbit polyclonal anti- SQSTM1/p62	Cell Signaling Technology	5114S<br>RRID:AB_10624872
Rabbit monoclonal anti- Atg7	Cell Signaling Technology	8558<br>RRID:AB_10831194
Rabbit Polyclonal anti-ULK1	Abways	CY6902
Rabbit Polyclonal anti- ADRP/Perilipin 2	Proteintech	15294-1-AP<br>RRID:AB_2878122
Rat monoclonal anti- LAMP2	Abcam	ab13524<br>RRID:AB_2134736
Rabbit polyclonal anti- Hsf1	Proteintech	51034-1-AP<br>RRID:AB_2120269
Rabbit monoclonal anti-Usp39	Abcam	ab131332<br>RRID:AB_11155124
Rabbit polyclonal to Usp39 - N-terminal	Abcam	ab236453
Rabbit Polyclonal anti-Usp39	Invitrogen	A304-816A<br>RRID:AB_2621011
Rabbit polyclonal anti-Sox9	Millipore	AB5535<br>RRID:AB_2239761
Mouse monoclonal anti-SC35	Abcam	ab11826<br>RRID:AB_298608
Rabbit polyclonal anti-Ki67	Abcam	ab15580<br>RRID:AB_443209
Rabbit polyclonal anti -PCNA	Servicebio	GB11010-1-100<br>RRID:AB_2811188
Rabbit polyclonal anti -F4/80	Servicebio	GB11027<br>RRID:AB_2814687
Rabbit polyclona anti-Caspase -3	Cell Signaling Technology	9662<br>RRID:AB_331439
Rabbit polyclona anti-Cleaved Caspase -3 (Asp175)	Cell Signaling Technology	9661<br>RRID:AB_2341188

Rabbit polyclonal anti-Lamin B1	Proteintech	12987-1-AP<br>AB_2136290
Rabbit polyclonal anti-GAPDH	Proteintech	10494-1-AP<br>RRID:AB_2263076
peroxidase AffiniPure Goat Anti-Rabbit IgG (H+L)	Jackson ImmunoResearch	111-035-144<br>RRID:AB_2307391
Peroxidase AffiniPure Goat Anti-Mouse IgG (H+L)	Jackson ImmunoResearch	115-035-003<br>RRID:AB_10015289
Bacterial and virus strains		
DH5α Chemically Competent Cell	Shanghai Weidi biotechnology Co.Ltd.	DL1001
Stbl3 Chemically Competent Cell	Shanghai Weidi biotechnology Co.Ltd.	DL1046
Biological samples		
NAFLD and NASH liver samples from human patients	Qilu Hospital of Shandong University	N/A
Chemicals, peptides, and recombinant proteins		
Isopropyl β-D-thiogalactoside	Sangon Biotech	A600168
TUNEL Assay Kit	Keygen	KGA7072
Penicillin-Streptomycin Solution, 100X	Biosharp	BL505A
Sodium pyruvate	Sigma	P4562
Insulin	Macgene	CC101
Insulin injection	FOSUN PHARMA	6939863702032
Dimethyl sulfoxide（DMSO）	Aladding	D103277
Doxycycline hydrochloride	Sangon Biotech	A600889
L- thyroxine	Sigma	T1775
Collagenase Type IV	Sigma	C5138
BODIPY	Cayman	25892
Oil Red O	Sigma	O0625
Dexamethasone	aladdin	D137736
EGTA	Solarbio	E8050
Tamoxifen	Sigma	T5648
Trypsin without ethylenediaminetetraacetic acid (EDTA)	Macgene	CC035
4% paraformaldehyde	Servicebio	G1101
ProLong™ Gold Antifade Mountant with DAPI	Thermo Fisher Scientific	P36941
Mounting Medium With DAPI - Aqueous, Fluoroshield	Abcam	ab104139
Mounting Medium, antifading	Solarbio	S2100
Hoechst 33342 Stain solution	Solarbio	C0031
ProLong™ Gold Antifade Mountant with DAPI	Thermo Fisher Scientific	P36941
Williams medium E	Thermo Fisher Scientific	12551032
Nicotinamide	Solarbio	N8070
L-Glutamine	Sangon Biotech	A600224
Glycerin Gelatin Mounting Tablets	Servicebio	G1402-30ML

Isoflurane	RWD life science Co.	R510-22-10
Research Diets D12492 60 kcal% Fat	Research Diets	D12492
α-D-glucose	Solarbio	G8150
Sucrose	Sangon Biotech	A100335-0250
Jetprime	polyplus-transfection	101000046
BODIPY 558/568 red c12	Cayman	27014
Lipofectamine® 2000 CD (Chemically Defined) Transfection Reagent	Thermo Fisher Scientific	12566014
Water Nuclease-Free	Servicebio	G4700
Critical commercial assays		
His-tag Protein Purification Kit	Beyotime	P2226
Cell Total RNA Isolation Kit	Foregene	RE-03111
Triglyceride assay kit	Nanjing jiancheng Bioengineering Institute	A110-1-1
Nonesterified Free fatty acids assay kit	Nanjing jiancheng Bioengineering Institute	A042-2-1
RNAMAX-T7 in vitro transcription kit	RiboBio	C11001-1
Picro Sirius Red Stain	Anhui Leagene Biotechnology Co., Ltd.	DC0041
Pierce™ RNA 3' End Desthiobiotinylation Kit	Thermo Fisher Scientific	20163
Magnetic RNA Protein Pull-Down Kit	Thermo Fisher Scientific	20164
Spin Column RNA Cleanup & Concentration Kit	Sangon	B518688
Nucleoprotein Extraction Kit	Sangon	C500009
EZ-Nuclear RIP Kit	Merck Millipore	17-701
Deposited data		
RNA sequencing (RNA-seq) of Usp39 knockout by in 5-weeks-old male mice	This paper	GSE213633
RNA immunoprecipitation sequencing (RIP-seq) of Usp39 in AML12 cells	This paper	GSE213629
Lipidomics Sequencing Data of Usp39 knockout by in 5-weeks-old male mice	This paper	N/A
Experimental models: Cell lines		
Mouse: AML12 alpha mouse liver 12 cell line	ATCC: The Global Bioresource Center	RRID:CVCL_0140
Mouse: Hepa1-6 Hepatoma epithelial cell line	Peihui-wang lab	RRID:CVCL_0327
Human: 293T Embryo kidney epithelial cell line	National collection of authenticated cell cultures	RRID:CVCL_0063
Human: Hepg2 hepatoma cell line	ATCC: The Global Bioresource Center	RRID:CVCL_0027
Human: Huh7 hepatoma cell line	National collection of authenticated cell cultures	RRID:CVCL_0336
Experimental models: Organisms/strains		
Mouse: Usp39$^{flox/flox}$	Gempharmatech	C57BL/6J mice

Mouse: Albumin-Cre	Gempharmatech	C57BL/6J mice
Oligonucleotides		
siRNA targeting sequence: Hsf1 #1: CCTGAAGAGTGAGGACATA	This paper	N/A
siRNA targeting sequence: Hsf1 #2: GGACACAACCGGAGCCCAA	This paper	N/A
siRNA targeting sequence: Usp39#1: GCATCAAGAGATTTACTAA	This paper	N/A
siRNA targeting sequence: Usp39#2: CAAGTGGTATGAATTACAA	This paper	N/A
PLKO.1-shUsp39#2-TRC shRNA targeting sequence: CCTGACAACTATGAAATCATT	WZ Biosciences	TRCN0000030927
PLKO.1-shUsp39#4-TRC shRNA targeting sequence: CCCGCTCTATAAGGATGAGAA	WZ Biosciences	TRCN0000030925
Primers for RNA-pull down: Hsf1-E5-I7-Forward TAATACGACTCACTATAGGGGAGAACGAGGCCCTGTGG	This paper	N/A
Primers for RNA-pull down: Hsf1-E5-I7- Reverse TGGGAGCCAGCTCAGTGATA	This paper	N/A
Primers for plasmid construction: Usp39-pT3G-3FPS-Forward ACGAAGGGCGGAGGAGGATCCATGTCTAGCCGGTCCAAGCG	This paper	N/A
Primers for plasmid construction: Usp39-pT3G-3FPS-Reverse-CCGTCATAGCGCGTTAATTAATCAAGCCCCCTGCTGGTT	This paper	N/A
Primers for plasmid construction: Hsf1-pLenti- C-Myc-DDK-IRES-Puro Forward AGGAGATCTGCCGCCGCGATCGCATGGATCTGGCCGTGGGC	This paper	N/A
Primers for plasmid construction: Hsf1-pLenti- C-Myc-DDK-IRES-Puro Reverse-CTCGAGCGGCCGCGTacgcgtGGAGACAGTGGGGTCCTTGG	This paper	N/A
Primers for RT-PCR, see Table S2	This paper	N/A
Primers for semiquantitative RT-PCR see Table S2		
Primers for RIP-qPCR, see Table S2	This paper	N/A
Recombinant DNA		

Doxycycline-inducible lentiviral vector pT3G-3FPS-Usp39	This paper	N/A
pLenti- C-Myc-DDK-IRES-Puro- Hsf1	This paper	N/A
pLenti- C-Myc-DDK-IRES-Puro vector	origene	
PLKO.1-TRC-shUsp39	WZ Biosciences	
pcDNA™3.1/myc-His A vector	Invitrogen™	
Software and algorithms		
FastQC v0.11.9	N/A	https://www.bioinformatics.babraham.ac.uk/projects/fastqc/
bowtie v1.2.3	Langmead et al.[1]	http://bowtie-bio.sourceforge.net/index.shtml
HISAT2 v2.2.0	Zhang et al.[2]	http://daehwankimlab.github.io/hisat2/
Samtools v1.9	Li et al.[3]	http://samtools.sourceforge.net/
featureCounts v2.0.0	Liao Y et al.[4]	http://subread.sourceforge.net/featureCounts.html
rMATS v4.1.0	Shen et al.[5]	http://rnaseq-mats.sourceforge.net/
HOMER v4.11	Heinz et al.[6]	http://homer.ucsd.edu/homer/
Gene Ontology	The Gene Ontology.[7]	http://geneontology.org/
GraphPad Prism 8	N/A	https://www.graphpad.com/scientific-software/prism/
ImageJ v2.1.0	Schneider et al.[8]	https://imagej.nih.gov/ij/
SPSS 24.0	N/A	https://www.ibm.com/cn-zh/spss
Seahorse Wave software	N/A	https://www.agilent.com.cn/zh-cn/product/cell-analysis/real-time-cell-metabolic-analysis/xf-software/seahorse-wave-desktop-software-740897
Integrative Genomics Viewer (IGV) v2.8.2	Robinson et al.[9]	https://igv.org/
Other		

Note: Primer sequences are all produced by Sangon Biotech (Shanghai), unless the manufacturer is specifically indicated in the table.

Reference

1. Langmead B, Trapnell C, Pop M, Salzberg SL. Ultrafast and memory-efficient alignment of short DNA sequences to the human genome. *Genome biology* **10**, R25 (2009).

2. Zhang Y, Park C, Bennett C. Rapid and accurate alignment of nucleotide conversion sequencing reads with HISAT-3N. **31**, 1290-1295 (2021).

3. Li H*, et al.* The Sequence Alignment/Map format and SAMtools. *Bioinformatics (Oxford, England)* **25**, 2078-2079 (2009).

4. Liao Y, Smyth GK, Shi W. featureCounts: an efficient general purpose program for assigning sequence reads to genomic features. *Bioinformatics (Oxford, England)* **30**, 923-930 (2014).

5. Shen S, Park JW, Lu ZX. rMATS: robust and flexible detection of differential alternative splicing from replicate RNA-Seq data. **111**, E5593-5601 (2014).

6. Heinz S*, et al.* Simple combinations of lineage-determining transcription factors prime cis-regulatory elements required for macrophage and B cell identities. *Molecular cell* **38**, 576-589 (2010).

7. The Gene Ontology Resource: 20 years and still GOing strong. *Nucleic acids research* **47**, D330-d338 (2019).

8. Schneider CA, Rasband WS, Eliceiri KW. NIH Image to ImageJ: 25 years of image analysis. *Nature methods* **9**, 671-675 (2012).

9. Robinson JT, Thorvaldsdóttir H, Wenger AM, Zehir A, Mesirov JP. Variant Review with the Integrative Genomics Viewer. *Cancer research* **77**, e31-e34 (2017).

Supplementary Table 4. Information for normal and NASH samples

Items	Sex	Age (Years)	BMI (Kg/m^2)	AST (U/L)	ALT (U/L)	TG (mmol/L)	TC (mmol/L)	FBG (mmol/L)	NAS
Non-steatosis	Male	50-60	27.2	28	29	1.75	5.03	4.69	0
Non-steatosis	Male	60-70	24	88	105	0.76	4.21	4.84	0
Non-steatosis	Male	40-50	27	19	23	1.42	4.25	4.68	0
Non-steatosis	Male	50-60	28.1	31	15	1	4.2	5.09	0
Non-steatosis	Male	40-50	31.6	21	15	0.99	6.8	5.67	0
NASH	Male	50-60	23.7	17	15	1.11	4.01	8.35	5
NASH	Male	30-40	34	72	125	2.21	5.67	8.9	2
NASH	Male	20-30	32	40	49	2.07	4.31	7.2	2
NASH	Female	30-40	35.6	25	20	0.95	3.9	5.21	2
