# Supplementary material for: Spliceosome component Usp39 contributes to hepatic lipid homeostasis through the regulation of autophagy
Source: Nat Commun. 2023 Nov 3;14:7032. doi: 10.1038/s41467-023-42461-6 (PMC10624899; doi:10.1038/s41467-023-42461-6)
Supplement: Supplementary file 3 — Description of Additional Supplementary Files [file 41467_2023_42461_MOESM3_ESM.pdf]

Description of Additional Supplementary Files

File Name: Supplementary Data 1

Description: Gene expression profile of Usp39 knockout mice

File Name: Supplementary Data 2

Description: Alternative splicing analysis in RNA-seq data of Usp39 knockout mice liver

File Name: Supplementary Data 3

Description: Combined analysis of RIP-seq and AS events reveals Usp39 regulated targets

File Name: Supplementary Data 4

Description: Lipidomics data of Usp39-HKO and control mice liver
