# Supplementary material for: Spliceosome component Usp39 contributes to hepatic lipid homeostasis through the regulation of autophagy
Source: Nat Commun. 2023 Nov 3;14:7032. doi: 10.1038/s41467-023-42461-6 (PMC10624899; doi:10.1038/s41467-023-42461-6)
Supplement: Supplementary file 8 — Reporting Summary [file 41467_2023_42461_MOESM8_ESM.pdf]

nature portfolio

Corresponding author(s): Zhaojian Liu

Last updated by author(s): Sep 26, 2023

Reporting Summary

Nature Portfolio wishes to improve the reproducibility of the work that we publish. This form provides structure for consistency and transparency in reporting. For further information on Nature Portfolio policies, see our Editorial Policies and the Editorial Policy Checklist.

Statistics

For all statistical analyses, confirm that the following items are present in the figure legend, table legend, main text, or Methods section.

n/a	Confirmed	
☐	☒	The exact sample size (*n*) for each experimental group/condition, given as a discrete number and unit of measurement
☐	☒	A statement on whether measurements were taken from distinct samples or whether the same sample was measured repeatedly
☐	☒	The statistical test(s) used AND whether they are one- or two-sided<br>*Only common tests should be described solely by name; describe more complex techniques in the Methods section.*
☒	☐	A description of all covariates tested
☐	☒	A description of any assumptions or corrections, such as tests of normality and adjustment for multiple comparisons
☐	☒	A full description of the statistical parameters including central tendency (e.g. means) or other basic estimates (e.g. regression coefficient) AND variation (e.g. standard deviation) or associated estimates of uncertainty (e.g. confidence intervals)
☐	☒	For null hypothesis testing, the test statistic (e.g. *F*, *t*, *r*) with confidence intervals, effect sizes, degrees of freedom and *P* value noted<br>*Give P values as exact values whenever suitable.*
☒	☐	For Bayesian analysis, information on the choice of priors and Markov chain Monte Carlo settings
☒	☐	For hierarchical and complex designs, identification of the appropriate level for tests and full reporting of outcomes
☐	☒	Estimates of effect sizes (e.g. Cohen's *d*, Pearson's *r*), indicating how they were calculated

Our web collection on statistics for biologists contains articles on many of the points above.

Software and code

Policy information about availability of computer code

Data collection	QuantStudio Design & Analysis software was used for qPCR data.<br>RIP-seq was sequenced by paired-end 150bp sequencing performed on the Illumina Novaseq 6000 platform.<br>RNA-seq data was generated by sequencing performed on theIllumina Novaseq 6000 platform.<br>Lipidomics adopts a non-targeted lipidomics analysis platform based on UPLC-Orbitrap mass spectrometry system, combined with LipidSearch software (Thermo Scientific) for lipid identification and data preprocessing.<br>Mitochondrial respiration was measurement by seahorse XFe9 (Agilent).<br>Electron microscopy imaged were obtained using a Hitachi HT7800 TEM.<br>Fluorescent images were captured on a Dragonfly 200 confocal microscopy system (Andor Technology).<br>Bright field images were obtained using an Olympus BX53 microscope system.<br>Columbus Instruments Comprehensive Lab Animal Monitoring System CLAMS6 was used to measure the metabolic rate. Data were generated by automatic recording of the instrument.
Data analysis	Agarose gel electrophores, Electron microscopy imaged, Fluorescent and Bright field images, is quantification : Fiji (version 1.53c).<br>For statistical analysis: SPSS 24.0 and GraphPad Prism (version 8).<br>The Seahorse Wave software and graphPad Prism (version 8) was used to analyze mitochondrial respiration measurement data.<br>Analyzing mouse metabolic rate data with GraphPad Prism (version 8).<br>RNA-seq reads were aligned to the mouse genome (mm10) with HISAT2 (version 2.2.0). Mapped reads were visualized with the Integrative Genomics Viewer (IGV). Gene expression quantification was performed by counting reads over genes from the same annotation as alignment using featureCounts version 2.0.0. Differential gene expression analysis was conducted using DESeq2. The cutoff was set p < 0.05 and log2 fold change (FC) > 0.5 or < -0.5. The mapped reads aligned by HISAT2 were further used for AS analysis and the events were identified with rMATS (version 4.1.0) (Shen et al. 2014).

Lipidomics adopts a non-targeted lipidomics analysis platform based on UPLC-Orbitrap mass spectrometry system, combined with LipidSearch software (Thermo Scientific) for lipid identification and data preprocessing. Precise and global identification of phospholipid molecular species by an Orbitrap mass spectrometer and automated search engine Lipid Search. J Chromatogr A. 2010, 1217 (25):4229-4239]. Principal component analysis (PCA) was used to evaluate overall distribution trend and the degree of variation of samples between groups. Differential lipid molecules were selected by Student's t-test ($p < 0.05$) and fold change (FC > 1.5 or FC < 0.67).
The combined lipidome and transcriptome analysis and p-values were calculated by IPA (Ingenuity Pathway Analysis).
For RIP-seq data, the adaptor sequences and low-quality reads were removed with Trim Galore (version 0.6.1), and the quality of the clean reads was checked with FastQC (version 0.11.9). rRNA sequences were removed with bowtie (version 1.2.3) (Langmead et al. 2009) , and the remaining reads were mapped to the mouse genome (mm10) with HISAT2 and visualized in IGV (Robinson et al. 2017). Usp39 binding peaks were identified with Piranha (-p 0.001 -z 100) and annotated with an R package clusterProfiler. De novo motif analysis was conducted with HOMER.

For manuscripts utilizing custom algorithms or software that are central to the research but not yet described in published literature, software must be made available to editors and reviewers. We strongly encourage code deposition in a community repository (e.g. GitHub). See the Nature Portfolio guidelines for submitting code & software for further information.

Data

Policy information about availability of data

All manuscripts must include a data availability statement. This statement should provide the following information, where applicable:

- Accession codes, unique identifiers, or web links for publicly available datasets
- A description of any restrictions on data availability
- For clinical datasets or third party data, please ensure that the statement adheres to our policy

The publicly available RNA-seq data sets of NAFLD and NASH mice liver are achieved from GEO database (GSE165855 and GSE154892).
The RNA-seq and RIP-seq data for this study are available for download from the Gene Expression Omnibus (GEO) repository(GSE213633 [https://www.ncbi.nlm.nih.gov/geo/query/acc.cgi?acc=GSE213633], GSE213629 [https://www.ncbi.nlm.nih.gov/geo/query/acc.cgi?acc=GSE213629]). All the two datasets were summarized and deposited at GEO database (https://www.ncbi.nlm.nih.gov/geo/query/acc.cgi?acc=GSE213635).
The raw data for lipidomics are available in Supplementary Data 4. The raw data are publicly available as of the date of publication.

Research involving human participants, their data, or biological material

Policy information about studies with human participants or human data. See also policy information about sex, gender (identity/presentation), and sexual orientation and race, ethnicity and racism.

Reporting on sex and gender	Sex and gender were not considered in study design.
Reporting on race, ethnicity, or other socially relevant groupings	N/A
Population characteristics	Human NASH clinical samples were obtained from the Department of Hepatobiliary Surgery, Qilu Hospital of Shandong University. Human liver tissue samples were collected painlessly from adult patients with NASH who underwent liver transplantation or liver biopsy. NASH liver samples were collected with a NAS ≥2, fibrosis was included in the NASH samples. Human healthy liver samples from patients who benign liver disease biopsy sample or benign neoplastic pathological changes. The characteristics for NASH patients were listed in Supplementary table S4.
Recruitment	Human healthy and NASH liver samples were from the Department of General Surgery, Shandong University, with participants gave written informed consent, according to CARE guidelines and in compliance with the Declaration of Helsinki principles. No compensation was provided to participants for this study. Human healthy liver samples from patients who benign liver disease biopsy sample or benign neoplastic pathological changes taken on the day of surgery. The study was approved by the Research Ethics Committee of Shandong University (SDULCLL2019-1-09). Sex was not considered in this study due to the low frequency of subjects. Sample size and complete randomization of selection may lead to bias on our study. To overcome the bias, ur data was validated by a large cohort of human NAFLD (GSE193084) sequencing data. Detailed characteristics of human healthy and NASH patients have been listed in Supplementary Table S4.
Ethics oversight	The protocols of our study were approved by the Ethics Committee of Shandong University (SDULCLL2019-1-09).

Note that full information on the approval of the study protocol must also be provided in the manuscript.

Field-specific reporting

Please select the one below that is the best fit for your research. If you are not sure, read the appropriate sections before making your selection.

☒ Life sciences ☐ Behavioural & social sciences ☐ Ecological, evolutionary & environmental sciences

For a reference copy of the document with all sections, see nature.com/documents/nr-reporting-summary-flat.pdf

Life sciences study design

All studies must disclose on these points even when the disclosure is negative.

Sample size	The sample size of each experiment is provided in the figure/table legends in the main manuscript and supplementary file. For RNA-seq, samples were prepared at least in four biological replicates. For liver electron microscope , samples were prepared at least in ten biological replicates. For RIP-seq, samples were prepared at three independent experiments. For animal experiments, each group has at least three mice. These sample sizes are sufficient to achieve statistical significance. Exact sample size are recorded in the paper. These sizes have previously been shown as sufficiently powered to determine statistical differences in mean values of our investigated parameters.
Data exclusions	No data was excluded from analysis, except for necessary data quality control step for initial RNA-seq and RIP-seq data processing.
Replication	The replication number is indicated in the legend of corresponding figures where applicable. All attempts at replication were successful.
Randomization	Cells and mice were randomly allocated into experimental groups. Randomized grouping is conducted using a computer program to generate pseudo-random numbers.
Blinding	Investigators were blinded to analysis whenever it is available. Blinding was used for animal allocation , data collection and analyses.

Reporting for specific materials, systems and methods

We require information from authors about some types of materials, experimental systems and methods used in many studies. Here, indicate whether each material, system or method listed is relevant to your study. If you are not sure if a list item applies to your research, read the appropriate section before selecting a response.

Materials & experimental systems

n/a	Involved in the study	
☐	☒	Antibodies
☐	☒	Eukaryotic cell lines
☒	☐	Palaeontology and archaeology
☐	☒	Animals and other organisms
☒	☐	Clinical data
☒	☐	Dual use research of concern
☒	☐	Plants

Methods

n/a	Involved in the study	
☒	☐	ChIP-seq
☒	☐	Flow cytometry
☒	☐	MRI-based neuroimaging

Antibodies

Antibodies used

Rabbit monoclonal anti-USP39 , Abcam, ab131244; RRID:AB_11155482
Mouse monoclonal anti-SC35, Abcam, ab11826; RRID:AB_298608
Rabbit monoclonal anti- LC3A/B (D3U4C) XP, Cell Signaling Technology ,12741; RRID:AB_2617131
Rabbit polyclonal anti- Albumin, proteintech ,16475-1-AP; RRID:AB_2242567
Mouse monoclonal anti-Actin , proteintech, 66009-1-Ig; RRID:AB_2687938
Rabbit polyclonal anti- SQSTM1/p62, Cell Signaling Technology, 5114; RRID:AB_10624872
Rabbit monoclonal anti- Atg7, Cell Signaling Technology, 8558; RRID:AB_10831194
Rabbit Polyclonal anti-ULK1 abways CY6902
Rabbit Polyclonal anti- ADRP/Perilipin 2, proteintech,15294-1-AP; RRID:AB_2878122
Rat monoclonal anti- LAMP2, Abcam, ab13524; RRID:AB_2134736
Rabbit polyclonal anti- HSF1 , proteintech, 51034-1-AP; RRID:AB_2120269
Rabbit monoclonal anti-Usp39 Abcam, ab131332 RRID:AB_11155124
Rabbit polyclonal to USP39 - N-terminal, Abcam, ab236453
Rabbit Polyclonal anti-Usp39, Invitrogen, A304-816A RRID:AB_2621011
Rabbit polyclonal anti-Ki67 , Abcam, ab15580 RRID:AB_443209
Rabbit polyclonal anti -PCNA, servicebio, GB11010-1-100 RRID:AB_2811188
Rabbit polyclonal anti -F4/80, servicebio, GB11027 RRID:AB_2814687
Rabbit polyclonal anti-SOX9, Millipore, AB5535 RRID:AB_2239761
Rabbit polyclona anti-Caspase-3, Cell Signaling Technology, 9662 RRID:AB_331439
Rabbit polyclona anti-Cleaved Caspase-3 (Asp175) , Cell Signaling Technology, 9661 RRID:AB_2341188
Rabbit polyclonal anti-Lamin B1, proteintech , 12987-1-AP AB_2136290
Rabbit polyclonal anti-GAPDH , proteintech,10494-1-AP RRID:AB_2263076
peroxidase AffiniPure Goat Anti-Rabbit IgG (H+L), Jackson ImmunoResearch , 111-035-144 RRID:AB_2307391
Peroxidase AffiniPure Goat Anti-Mouse IgG (H+L),Jackson ImmunoResearch, 115-035-003 RRID:AB_10015289

Validation

The antibodies used in this study are commercially available and validated by the manufacture. Based on the catalog number, the information of these antibody, including validation statements, relevant citations and antibody profiles in online databases, is available in the manufacture's website.

Rabbit monoclonal anti-USP39, Abcam, ab131244 WB IF https://www.abcam.cn/usp39-antibody-epr8683-ab131244.html
Mouse monoclonal anti-SC35, Abcam, ab11826 IF https://www.abcam.cn/products/primary-antibodies/sc35-antibody-sc-35-nuclear-speckle-marker-ab118.
Rabbit monoclonal anti- LC3A/B (D3U4C) XP, Cell Signaling Technology, 12741 WB https://www.cellsignal.cn/products/primary-antibodies/lc3a-b-d3u4c-xp-rabbit-mab/12741
Rabbit polyclonal anti- Albumin, proteintech, 16475-1-AP IF https://www.ptgcn.com/products/ALB-Antibody-16475-1-AP.htm
Mouse monoclonal anti-Actin, proteintech, 66009-1-Ig WB https://www.ptgcn.com/products/Pan-Actin-Antibody-66009-1-Ig.htm
Rabbit polyclonal anti- SQSTM1/p62, Cell Signaling Technology, 5114 WB https://www.cellsignal.cn/products/primary-antibodies/sqstm1-p62-antibody/5114
Rabbit monoclonal anti- Atg7, Cell Signaling Technology, 8558 WB https://www.cellsignal.cn/products/primary-antibodies/atg7-d12b11-rabbit-mab/8558
Rabbit Polyclonal anti-ULK1, abways, CY6902 WB http://www.abways.cn/showproduct.asp?cid=CY6902
Rabbit Polyclonal anti- ADRP/Perilipin 2, proteintech, 15294-1-AP IF https://www.ptgcn.com/products/ADRP-Antibody-15294-1-AP.htm
Rat monoclonal anti- LAMP2 Abcam, ab13524 IF https://www.abcam.cn/lamp2-antibody-gl2a7-ab13524.html
Rabbit polyclonal anti- HSF1, proteintech, 51034-1-AP WB https://www.ptgcn.com/products/HSF1-Antibody-51034-1-AP.htm
Rabbit monoclonal anti-Usp39 Abcam, ab131332 WB IHC https://www.abcam.cn/products/primary-antibodies/usp39-antibody-epr8684-ab131332.html
Rabbit polyclonal to USP39 - N-terminal, Abcam, ab236453 WB https://www.abcam.cn/products/primary-antibodies/usp39-antibody-n-terminal-ab236453.html
Rabbit Polyclonal anti-Usp39, Invitrogen, A304-816A WB https://www.thermofisher.cn/cn/zh/antibody/product/USP39-Antibody-Polyclonal/A304-816A
Rabbit polyclonal anti-Ki67, Abcam, ab15580 IHC https://www.abcam.cn/products/primary-antibodies/ki67-antibody-ab15580.html
Rabbit polyclonal anti -PCNA, servicebio,GB11010-1-100 IHC https://www.servicebio.cn/goodsdetail?id=1317
Rabbit polyclonal anti -F4/80, servicebio, GB11027 IHC https://www.servicebio.cn/goodsdetail?id=1330
Rabbit polyclonal anti-SOX9, Millipore, AB5535 IF https://www.merckmillipore.com/CN/zh/product/Anti-Sox9-Antibody,MM_NF-AB5535
Rabbit polyclona anti-Caspase-3, Cell Signaling Technology, 9662 WB https://www.cellsignal.cn/products/primary-antibodies/caspase-3-antibody/9662
Rabbit polyclona anti-Cleaved Caspase-3 (Asp175) ,Cell Signaling Technology, 9661 WB https://www.cellsignal.cn/products/primary-antibodies/cleaved-caspase-3-asp175-antibody/9661
Rabbit polyclonal anti-Lamin B1, proteintech , 12987-1-AP WB https://www.ptgcn.com/products/LMNB1-Antibody-12987-1-AP.htm
Rabbit polyclonal anti-GAPDH, proteintech,10494-1-AP WB https://www.ptgcn.com/products/GAPDH-Antibody-10494-1-AP.htm
peroxidase AffiniPure Goat Anti-Rabbit IgG (H+L) Jackson ImmunoResearch 111-035-144 WB https://www.jacksonimmuno.com/catalog/products/111-035-144
Peroxidase AffiniPure Goat Anti-Mouse IgG (H+L), Jackson ImmunoResearch, 115-035-003 WB https://www.jacksonimmuno.com/catalog/products/115-035-003

Eukaryotic cell lines

Policy information about cell lines and Sex and Gender in Research

Cell line source(s)	The AML12 (mouse liver cell line) (CRL-2254, USA) and HepG2 (HB-8065, USA) was from the ATCC,and the Hepa1-6 were a generous gift from Prof Peihui Wang, Advanced Medical Research Institute, Shandong University. HEK293T (SCSP-502, China) and Huh-7 (SCSP-526, China) cell line was obtained from the National Collection of Authenticated Cell Cultures.
Authentication	Cell lines were validated by STR profiling.
Mycoplasma contamination	All cell lines tested negative for mycoplasma contamination.
Commonly misidentified lines (See ICLAC register)	None.

Animals and other research organisms

Policy information about studies involving animals; ARRIVE guidelines recommended for reporting animal research, and Sex and Gender in Research

Laboratory animals	Usp39fl/+ and Albumin-Cre mice were obtained from GemPharmatech Company, and UBC-CreERT2 mice were obtained from the Jackson Laboratory. The Usp39-floxed mouse line was generated by flanking exon 2 of Usp39 with loxP sites. To generate hepatic-specific Usp39-HKO mice, Usp39fl/fl mice were crossed to the Albumin-Cre mouse line. A tamoxifen-inducible Usp39 knockout mice model (Usp39fl/fl; UBC-CreERT2) was also generated. All animal experiments were conducted in accordance with the guidelines of the Animal Care and Use Committee at Shandong University. All animals were housed in a constant temperature(21°C–22°C), humidity (60%±10% relative humidity) and pathogen-free-controlled environment cage with controlled lighting regime (12 h light; 12 h darkness) with freely available food and water.
Wild animals	No wild animals were used in these studies.
Reporting on sex	Sex was not considered in this animal experiments. Both male and female were used in the study.

Field-collected samples	No field collected samples in this study.
Ethics oversight	The Shandong University Animal Ethics Research Board approved the animal experiment procedures (ECSBMSSDU2022-2-113).

Note that full information on the approval of the study protocol must also be provided in the manuscript.
